# Insulin Metabolism in Polycystic Ovary Syndrome: Secretion, Signaling, and Clearance

**DOI:** 10.3390/ijms24043140

**Published:** 2023-02-05

**Authors:** Rok Herman, Jaka Sikonja, Mojca Jensterle, Andrej Janez, Vita Dolzan

**Affiliations:** 1 Department of Endocrinology, Diabetes and Metabolic Diseases, University Medical Centre Ljubljana, 1000 Ljubljana, Slovenia; 2 Department of Internal Medicine, Faculty of Medicine, University of Ljubljana, 1000 Ljubljana, Slovenia; 3 Pharmacogenetics Laboratory, Institute of Biochemistry and Molecular Genetics, Faculty of Medicine, University of Ljubljana, 1000 Ljubljana, Slovenia

**Keywords:** polycystic ovary syndrome, PCOS, insulin resistance, beta cell function, insulin clearance, glucose homeostasis, insulin signaling pathway

## Abstract

Polycystic ovary syndrome (PCOS) is the most common endocrine and metabolic disorder in women of reproductive age. Its heterogeneous clinical presentation is characterized by hyperandrogenemia, reproductive changes, polycystic ovary morphology, and insulin resistance (IR). The primary pathophysiological process in its multifactorial etiology has not yet been identified. However, the two most proposed core etiologies are the disruption of insulin metabolism and hyperandrogenemia, both of which begin to intertwine and propagate each other in the later stages of the disease. Insulin metabolism can be viewed as the interconnectedness of beta cell function, IR or insulin sensitivity, and insulin clearance. Previous studies of insulin metabolism in PCOS patients have yielded conflicting results, and literature reviews have focused mainly on the molecular mechanisms and clinical implications of IR. In this narrative review, we comprehensively explored the role of insulin secretion, clearance, and decreased sensitivity in target cells as a potential primary insult in PCOS pathogenesis, along with the molecular mechanism behind IR in PCOS.

## 1. Introduction

Polycystic ovary syndrome (PCOS) is the most common endocrine/metabolic disorder in women of reproductive age. It affects up to 20% of women worldwide, and its prevalence has been on the rise over the last decade [1]. Its development and clinical presentation are characterized by multiple underlying metabolic, hyperandrogenic, and reproductive abnormalities. Despite the continuous efforts to identify the primary pathophysiological process, the scientific community is still torn between different proposed theories, and the precise and unifying mechanism remains to be identified. Consequently, the treatment, to a large degree, still depends on lifestyle intervention and symptomatic management of individual signs and symptoms. The new era of anti-obesity therapy offers a novel powerful pharmacologic tool to improve metabolic, reproductive, and other clinical outcomes in a subset of patients who are overweight or obese, primarily through weight reduction [2]. However, a better understanding of individual patient phenotypes and the dominant pathophysiological process is necessary to improve the management of all patients.

Three diagnostic criteria sets are commonly used to divide the syndrome into four distinct phenotypes [3]. Heterogeneous clinical presentation, combined with numerous vicious cycles and complex multidirectional relationships in its pathophysiology, add to the difficulty of pinpointing the origin of this process. Over the years, the two most commonly proposed core etiologies have been: (i) insulin resistance (IR), with associated hyperinsulinemia, and (ii) hyperandrogenemia. Both begin to notably intertwine and propagate each other in the later stages of the disease [4]. In addition, a significant proportion of patients suffer from excess weight, which is associated with worse clinical presentation and contributes to both IR and hyperandrogenemia through well-documented pathways. Newer concepts, including developmental programming by environmental or hormonal factors and the effects of gut microbiota dysbiosis, are becoming increasingly recognized and investigated [5,6]. There is, however, a lack of longitudinal data regarding how the clinical picture and its metabolic and endocrinological symptoms progress over the patients’ lifetime, which could aid in the understanding of the syndrome.

In this narrative review, we comprehensively explore the role of abnormal insulin levels and decreased insulin sensitivity in target cells as the potential primary insult in PCOS pathogenesis. We also cover the underlying molecular mechanisms of IR and its relationship with dysfunctional insulin secretion from pancreatic beta cells and hepatic insulin clearance, resulting in a range of metabolic abnormalities from disproportionate hyperinsulinemia to glucose intolerance and type 2 diabetes (T2D).

## 2. The Role of Insulin Resistance in PCOS

The coexistence of IR and PCOS was first described in 1980, when it was demonstrated that hyperandrogenism correlates with hyperinsulinism in women with PCOS [7]. A few years later, a small study found that even lean patients with PCOS are insulin resistant [8], which paved the way to the hypothesis that IR might be central to PCOS. Despite this finding, the established PCOS diagnostic criteria continued to focus on hyperandrogenism and ovarian morphology and function, without taking into account the clinical variables related to glucose metabolism. Consequently, a significant degree of metabolic heterogeneity is observed between the four phenotypes, based on Rotterdam diagnostic criteria, with IR being the most dominant proposed etiology in the subgroup of phenotype A patients [9]. The current estimates are that up to 75% of PCOS women have impaired insulin response, as measured by the hyperinsulinemic-euglycemic clamp method [10]. Despite numerous studies investigating the relationship between excess weight, decreased insulin sensitivity, and hyperinsulinemia in women with PCOS, our understanding of this multidirectional and synergistic web of interactions remains unclear [11]. An additional degree of uncertainty comes from the frequent use of different surrogate estimates of IR and less convincing results for the degree of IR in lean PCOS patients in comparison to age- and BMI-matched controls [11,12,13,14,15,16].

Similar to T2D, in PCOS, the most commonly proposed sequence of events starts with IR as the primary insult, leading to compensatory hyperinsulinemia that temporarily maintains normal glycemia. However, with disease progression, relative or absolute insulin deficiency presents as prediabetes or T2D in the predisposed patients [10]. Throughout this process, a considerable period in the disease course is marked by supraphysiologic insulin levels that directly and indirectly disrupt ovarian function, as well as exert other, not yet fully explored, changes [9]. Insulin is able to act synergistically with luteinizing hormone as a co-gonadotrophin within ovarian theca cells by enhancing the production of androgens. Moreover, insulin mediates follicular development, promoting the arrest of pre-antral follicle development in the setting of hyperinsulinemia [9,17,18]. The clinical presentation of PCOS patients is influenced by other insulin related systemic effects through modulation of the luteinizing hormone pulse amplitude, stimulation of adrenal androgen secretion, and the suppression of hepatic sex hormone binding globulin production, which increases the amount of free testosterone [9,17,19,20].

As with other insulin-resistant conditions, it remains unresolved whether the decreased insulin activity might be due to an intrinsic defect in the insulin signaling pathway, or is instead induced by environmental factors, which further prevent the evaluation of IR in PCOS, as well as the development of causal interventions [21]. Still, the improvement in insulin sensitivity remains one of the primary desired treatment outcomes in most interventions, since extensive data reinforce the correlation between improved insulin sensitivity and other beneficial metabolic and reproductive outcomes [22,23]. Despite this fact, interventions that specifically targeted the insulin signaling pathway and directly enhanced insulin sensitivity have, to date, provided only limited clinical efficacy. In addition, although the initial studies in the 1980s showed increased levels of IR in lean PCOS patients, many conflicting results were later reported, and the described prevalence of IR is generally lower than that in overweight or obese patients [24]. In light of those questions and the importance of hyperinsulinemia alone in PCOS development, it is also essential to mention the potential conceptual shift in our understanding of IR proposed in recent years by some authors and research groups [25,26,27,28,29,30]. However, the amount of further data supporting this viewpoint is limited.

The underlying idea behind this view is that the regulation of insulin sensitivity is an integral component of normal metabolic physiology, as seen with the fluctuation in IR during illnesses, starvation, and pregnancy, which can be viewed as a homeostatic mechanism enabling sufficient nutrient supply to key tissues in diverse conditions [25,29]. Thus, the frequently observed IR in the pathogenesis of metabolic diseases could be seen as a protective cell mechanism against glucolipotoxicity in the state of constant positive energy balance by limiting the entry of excess glucose in the cells at times of concomitant high free fatty acid availability [25]. These mechanisms could, over the short term, be protective against pathological intracellular substrate accumulation in critical tissues, especially the cardiovascular system, but harmful in the medium to long term due to the systemic effects of IR [25,27]. The attempts to aggressively lower glycemia by overcoming IR might, in that view, force even more nutrients inside the cells, resulting in insulin-induced metabolic stress; therefore, nutrient restriction could be more beneficial than overriding IR alone [25].

The mitochondria are suggested to play a central role in the regulation of IR and ATP levels [25,30]. In the presence of excess energy, seen in obese patients, ATP production is elevated in insulin-sensitive cells, independent of energy demand, leading to mitochondrial overheating. ATP overproduction can potentially lead to IR through multiple mechanisms, such as the inhibition of AMPK, the induction of mTOR, and mitochondrial dysfunction, representing a feedback regulation of energy oversupply [30]. ATP overproduction is also a potential risk factor for insulin hypersecretion in beta cells, as well as glucagon secretion in alfa cells [30]. Some authors have also proposed that the hyperresponsiveness of beta cells to an obesogenic environment, such as a Westernized lifestyle, could be positioned as a primary insult, and that the hyperinsulinemia then drives IR, weight gain, and subsequent beta cell failure and development of T2D [29]. Some relevant studies to date have already suggested the idea that hyperinsulinemia and IR may represent two distinct features of the insulin metabolism dysregulation in PCOS [31]. However, despite some data supporting insulin hypersecretion resulting in hyperinsulinemia disproportionate to the level of IR in PCOS women [15,32,33,34], there are no PCOS studies applying this changed viewpoint to the study design, thus making any conclusions impossible. This viewpoint could have profound implications for the management of insulin-resistant conditions and PCOS, since restoring whole-body energy balance and the control of mitochondrial overheating could support strategies targeting caloric excess per se. The highest treatment efficacy in PCOS has been seen from interventions targeting this specific problem, such as lifestyle intervention through reduced caloric intake and ATP depletion by physical exercise, the effects on satiety through GLP-1 receptor agonists and bariatric surgery, or the reduction in ATP production by targeting the mitochondrial respiratory chain through metformin [25,30]. The hypothesis of the importance of hyperinsulinemia is also supported by studies demonstrating around a threefold higher prevalence of PCOS in type 1 diabetes patients. The proposed pathophysiological mechanism is that women with type 1 diabetes require supraphysiological doses of subcutaneous insulin to reach the required concentrations at the portal level. The intensive insulin therapy was suggested as one of the main reasons for the rising prevalence of PCOS in that specific group [35,36,37].

In addition, the assessment of the direct effects of hyperandrogenemia on IR and beta cell function is vital for its proper positioning in PCOS. Two studies carried out using the hyperinsulinemic-euglycemic clamp method showed a rapid onset of IR after supraphysiological androgen administration in women [38,39], and that IR can be partially reversed by androgen suppression or antiandrogen treatment [40,41].

Further perspective on the pathogenesis of IR in PCOS could also be gained from a few studies that, besides demonstrating significant differences in the gut microbiome profile between PCOS women and healthy controls, also found that the presence or absence of IR in PCOS patients significantly affected the microbiome [6,42,43,44,45,46]. The proposed theory is that the dysbiosis of gut microbiota leads to increased intestinal permeability and chronic low-grade inflammation, with a proinflammatory cytokine profile that can drive both IR and hyperinsulinemia [6]. Animal and human studies support the crossover effect behind the insulin receptor and the signal transduction of chronic subclinical inflammation, as well as the association between endotoxemia and chronic inflammatory response [46]. Besides the data indicating that microbiome composition can mediate the synthesis and secretion of insulin from beta cells, there is also some data that it affects androgen metabolism and follicular development [46]. It is also vital to take into account that hyperandrogenemia has multiple direct effects on intestinal microbiota composition [5,6].

**Figure 1:**
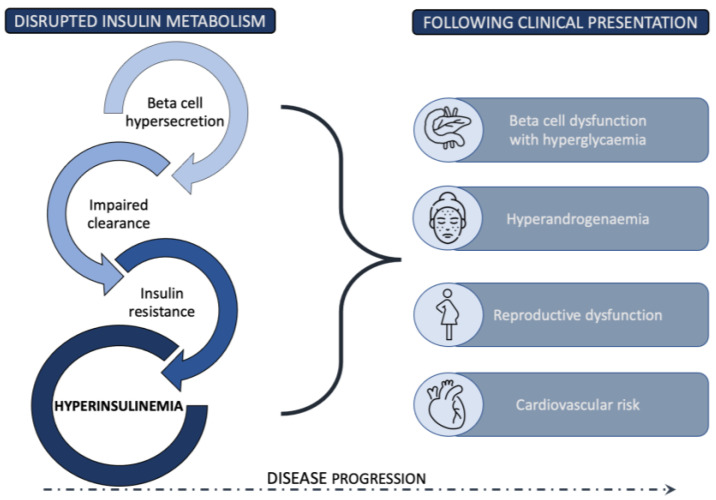
Disrupted insulin metabolism resulting in hyperinsulinemia as an essential pathophysiological driver in PCOS development.

The complex position of IR in PCOS pathophysiology and clinical presentation requires a comprehensive analysis of the entire insulin metabolism and its function at different stages. In the following sections, the steps leading from deviations in insulin secretion from pancreatic beta cells, to the mechanisms of impaired insulin action at the target cells, to the reduced insulin clearance are explored in detail. Figure 1 presents a summary of a new view of the role of insulin metabolism in PCOS pathogenesis, with hyperinsulinemia proposed as an important factor in PCOS development.

## 3. Pancreatic Beta Cell Function and PCOS

Studies have provided controversial results regarding the secretory function of pancreatic beta cells in PCOS patients. Some authors reported defective insulin secretion, whereas others demonstrated increased insulin secretion; however, in general, the data is limited. These discrepancies can, to some extent, be explained by different study protocols, in which research groups reported levels of insulin secretion in either basal or stimulated states, and only some study protocols adjusted the levels of insulin secretion to the prevailing level of IR. An essential aspect when extrapolating insulin levels to the estimates of beta cell function is that the role of insulin clearance also needs to be addressed. In addition, the way in which different stages of PCOS progression are characterized by diverse disturbances in glucose homeostasis is seldom assessed.

The occurrence of hyperinsulinemia in PCOS has previously been confirmed by the hyperinsulinemic clamp method. Interestingly, the same method demonstrated the presence of hyperinsulinemia in lean PCOS patients with normal insulin sensitivity, and even higher insulin secretion in lean patients than in obese patients or controls [15,16]. Furthermore, a comparison between PCOS patients and weight-matched controls demonstrated that PCOS patients had higher basal and cumulative 24 h insulin concentrations, despite having similar glucose concentrations. In contrast, their incremental insulin response to meals was markedly reduced, and further analysis showed that this reduction resulted from a decrease in amplitude, rather than pulse frequency [33].

In a more recent study, Pande et al. investigated metabolic features, including IR and beta cell function, in PCOS by including lean PCOS patients and comparing their data with those of obese PCOS patients and age- and BMI-matched controls. Beta cell activity was the highest in the lean PCOS group; however, no difference was observed between the other two groups [47]. A study by Manco et al. also supports the thesis that women with PCOS have insulin hypersecretion in comparison to appropriately matched IR controls [34]. When comparing their insulin metabolism with that of the IR-matched controls higher fasting and total insulin secretion was observed in PCOS patients than that of their peers [34]. In addition, Song et al. evaluated IR and beta cell function in euglycemic women with PCOS and compared the results to age- and BMI-matched healthy women. Women with PCOS showed higher IR and beta cell function than the controls, but not when lean patients were compared to matched controls separately [48].

On the other hand, by comparing normal-weight women with PCOS and no family history of T2D with normally ovulating women, matched for age and BMI, insulin sensitivity and insulin secretion were similar between two groups [49]. Ehrmann et al. found that during a frequently sampled IV glucose tolerance test, women with PCOS exhibit normal first-phase insulin secretion in absolute terms compared to women without PCOS. However, when first-phase insulin secretion was analyzed by taking into account either the degree of IR or basal insulin secretion, they found that women with PCOS who have a family history of diabetes were significantly more likely to demonstrate impairments in beta cell function [50]. Furthermore, the cross-sectional study involving Chinese women with PCOS and healthy controls detected an early impaired beta cell function in both lean and obese PCOS patients. However, a more serious primary defect in insulin action was detected in lean compared to obese patients [51]. In addition, Colilla et al. disclosed a heritable component to beta cell dysfunction in families of women with PCOS by comparing women with PCOS to their non-diabetic first-degree relatives. The authors demonstrated no evidence of sibling correlation in IR; however, impaired beta cell secretion was significantly correlated between both groups, as well as the parameter quantitating insulin secretion in relation to insulin sensitivity [52]. Similarly, one study also found unexpectedly low insulin secretion when corrected for peripheral insulin sensitivity in brothers of women with PCOS compared to control men [53].

Two studies provided comprehensive assessments of various contributing factors to insulin levels and IR in PCOS. In 2000, Morin-Papunen studied the contributions of body mass, body fat distribution, and family history of T2D to hyperinsulinemia, insulin secretion, and resistance in PCOS. The study compared lean and obese healthy controls with lean and obese women with PCOS. A trend towards hyperinsulinemia and impairment of insulin sensitivity was observed in lean PCOS subjects, but these changes were only significant in obese PCOS patients [17]. A study by Goodarzi et al. reported data from patients with PCOS who had undergone an assessment of IR, pancreatic beta cell function, obesity, and androgen levels. Multiple regression analysis clarified the phenotypic relationships, demonstrating that (i) IR and bioavailable testosterone were independent predictors of beta cell function; (ii) beta cell function and obesity were independent predictors of IR; and (iii) beta cell function was an independent predictor of bioavailable testosterone. Of note, comparison with healthy women revealed a significantly more robust relationship between beta cell function and IR in PCOS, raising the possibility of an intrinsic defect in beta cell function whereby increasing IR leads to greater than normal insulin response in PCOS. Coupled with the fact that beta cell function, not IR, was a predictor of hyperandrogenemia suggests that beta cell function may be a critical pathogenic determinant in PCOS [54]. Further perspective can be gained from the study in which exogenous androgenic steroids were applied to pregnant sheep, and fetal and postnatal pancreatic function and structure were examined. Beta cell numbers were significantly elevated in prenatally androgenized female fetuses, whereas alpha cell counts were unaffected, precipitating decreased alpha:beta cell ratios in the developing fetal pancreas, persisting into adolescence. In adolescence, basal insulin secretion was significantly higher in female offspring from androgen-excess pregnancies, and an exaggerated hyperinsulinemic response to glucose challenge was observed in the absence of IR, potentially implicating beta cells as a possibly primary locus of downstream metabolic perturbations [55]. This data supports the group’s previous findings that in the ovine and rhesus monkey model, abnormal pancreatic function likely precedes the development of IR and places hyperinsulinemia upstream [56,57].

To summarize, the available studies offer conflicting and limited results regarding the beta cell function in PCOS patients and provide little insight into the proper position of this function in PCOS pathogenesis. Heterogeneity in study designs including patients in different stages of the PCOS course; measurements of either basal, stimulated, or cumulative insulin concentrations; and not adjusting for the degree of IR and insulin clearance add to the difficulty of analyzing this topic. However, based on the current data, the role of beta cell function in the development of PCOS and its clinical presentation deserves further investigation.

## 4. Mechanisms of Insulin Resistance

IR is defined by an impaired response to insulin in target tissues—muscle, fat, and liver [58]—and predominantly manifests with a decreased utilization of glucose due to a defective glucose transport across the plasma membrane, facilitated by glucose transporter type 4 (GLUT4) [59]. IR in PCOS is the result of a post-receptor abnormality due to a disruption in signal transmission downstream from the insulin receptor. Thus, insulin-resistant tissues exhibit decreased responsiveness and sensitivity to insulin stimulation, whereas this effect is more pronounced in PCOS patients than in obese patients [60,61]. Apart from the metabolic effects of insulin, such as increased glucose uptake, glycogen synthesis, and protein synthesis, which are mediated through the phosphoinositide 3-kinase (PI3K) pathway, it also exhibits mitogenic and steroidogenic effects that are conveyed through the mitogen-activated protein kinase (MAPK) pathway. In patients with PCOS, IR selectively affects only the PI3K pathway, while the MAPK pathway functions normally [62,63]. Previous studies of IR mechanisms in common insulin-resistant states, such as obesity, T2D, and PCOS, have implicated that the pathogenesis of IR in the latter state might be unique [58,60,62,64]. In this chapter, we only briefly explore the molecular mechanisms associated with IR in PCOS. Since some of the presented findings had been provided by in vivo or in vitro studies on non-human models, translation to the human organism is somewhat limited. In addition, conflicting results from studies in different human body tissues indicate that the insulin signaling and molecular mechanisms behind IR may differ between tissues, restricting generalization.

**Figure 2:**
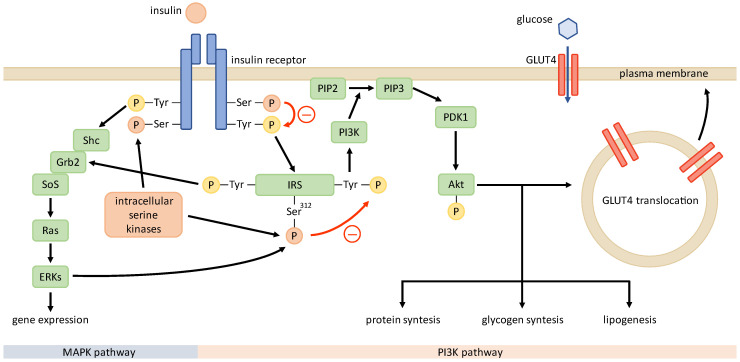
Insulin signaling pathway in PCOS. Unknown intracellular serine kinases may phosphorylate serine residues of insulin receptor and IRS, impairing the phosphorylation of tyrosine residues and signal transduction after stimulation with insulin. This renders the metabolic PI3K pathway defective, while the mitogenic MAPK pathway functions normally.

Insulin conveys its effects through the insulin signaling pathway (Figure 2). It starts with insulin binding to the insulin receptor and is followed by the autophosphorylation of the receptor’s tyrosine residues. This leads to the phosphorylation of the insulin receptor substrate (IRS) that conveys the signal downstream through the PI3K pathway and the MAPK pathway. PI3K drives the activation of protein kinase B (PKB or Akt), which interacts with numerous downstream proteins responsible for the metabolic effects of insulin [59], including the translocation of GLUT4 [65].

Adipocytes from PCOS patients exhibit a normal number and affinity of insulin-binding sites on their cell surface, indicating a defect downstream from the insulin receptor [60,66]. The constitutive phosphorylation of serine residues of the beta-subunit of the insulin receptor and IRS-1 Ser^312^, observed in higher levels in tissues of PCOS patients, impede the tyrosine phosphorylation of both the insulin receptor and IRS-1 after insulin stimulation [67,68]. The pretreatment of fibroblasts from PCOS patients with serine kinase inhibitors enhanced the tyrosine autophosphorylation of the insulin receptor [69], suggesting that a currently non-identified entity with serine kinase activity is involved in the process [60]. Furthermore, the phosphorylation of Ser^312^ in IRS-1 could be caused by the increased activity of intracellular kinases associated with the mitogenic MAPK pathway [62,63]. When looking at the signaling downstream of IRS, IRS-1-associated activation of PI3K was decreased in the skeletal muscles of women with PCOS [70]. Impaired PI3K activity may lead to a decreased activation of PKB, the main enzyme for propagating the translocation of GLUT4 to the cell membrane, resulting in reduced insulin-induced glucose uptake in target tissues [71]. Insulin is closely interconnected to the mitochondria, which are involved in regulation of cellular ATP and reactive oxygen species levels [72]. Impaired mitochondrial function in PCOS could be caused by the downregulation of nuclear-encoded genes involved in oxidative phosphorylation, leading to an increase in the production of reactive oxygen species [73,74]. Increased oxidative stress could then activate intracellular kinases, leading to the phosphorylation of serine residues of both the insulin receptor and IRSs, thus decreasing insulin sensitivity [75].

Initial genetic studies investigated the impact of individual genes, including those related to glucose metabolism, the insulin signaling pathway, and steroidogenesis, but did not yield firm evidence for the genetic etiology of PCOS, which is also influenced by environmental factors [76,77]. Several genome-wide association studies have been performed to explore the genetic architecture of PCOS [77,78,79,80,81,82], and the results showed that genes associated with gonadotropin function may be involved in PCOS development, and that genetic variants associated with PCOS are shared between ethnic groups [77,82]. While the National Institutes of Health criteria for PCOS are associated with the highest risk for IR and disruptions of glucose homeostasis compared to the Rotterdam criteria, a genome-wide association study showed that both criteria share most of the currently described susceptibility loci for PCOS, and that these results do not explain the differences in metabolic disturbances [83]. However, extensive genome-wide association studies exploring the genetic background between the individual PCOS phenotypes are lacking.

In summary, data from previous studies assessing the molecular mechanisms provided evidence that IR in PCOS could result from defective insulin signaling at various steps downstream from the insulin receptor. It seems that the phosphorylation of serine residues of insulin receptor and IRSs holds a central role in the pathogenesis of IR; however, the exact initial trigger remains to be identified.

## 5. Insulin Clearance in PCOS

When analyzing the relationship between hyperinsulinism and IR in PCOS, an essential and often overlooked aspect is the influence of the insulin clearance rate on insulin levels [31]. Since hyperinsulinemia plays a crucial role in the development and clinical presentation of PCOS, it is essential to acknowledge that plasma insulin levels reflect both the insulin secretion rate from beta cells and the metabolic clearance rate of insulin (MCRI) from plasma [21]. MCRI is determined by the efficiency of the insulin degradation process, which involves the internalization of the insulin-receptor complex and is followed by insulin degradation in the cytoplasm, carried out by insulin-degrading enzyme and other lysosomal enzymes [84,85]. Although there is a fair amount of data on the degree and prevalence of IR in women with PCOS, as well as some information on insulin secretion, MCRI was rarely investigated [21]. Most studies that have addressed this issue were performed in the 1980s–1990s and were relatively small. Newer and larger studies using more specific methods evaluating the potential impact of the patients’ PCOS phenotype would add to our current understanding of insulin metabolism in women with PCOS [31]. Along with the interpretation of peripheral levels of insulin that are most often used in the evaluation of insulin secretion and in the estimation of the degree of IR, it is essential to take into account that peripheral insulin levels do not always provide an appropriate approximation of portal insulin levels and do not elucidate the potential effect of the liver on glucose metabolism [31]. The large extent of inter- and intra-individual variability in hepatic insulin clearance has already been demonstrated [86,87,88,89].

Early studies made an important observation that the insulin-degrading enzyme has multiple cellular functions in addition to insulin degradation, including potential regulatory effects on the activity of glucocorticoid and androgen receptors [90,91]. Based on the noted discrepancy between serum insulin and C-peptide levels in some hyperandrogenic women with PCOS, in 1994, Buffington et al. hypothesized a possible association between testosterone and insulin metabolism. In their study, they found that the C-peptide to insulin ratio at baseline and the T-lymphocyte insulin degradation in the PCOS group to be twofold below those of the lean controls and weight-matched controls. Furthermore, basal C-peptide to insulin ratios and insulin-degradative activities were significantly and negatively interrelated, and both those parameters were highly correlated with basal testosterone levels [92]. In 1997, an important and well-designed study by Ciampelli et al. included 35 women with PCOS and 10 lean normal-ovulatory controls divided into four groups, according to their BMI and insulin secretion, who underwent OGTT and were monitored by the euglycemic-hyperinsulinemic clamp method. Women in the PCOS groups showed significantly higher insulin and C-peptide concentrations than did the controls. All the hyperinsulinemic PCOS patients had lower values of hepatic insulin clearance, independent of BMI, when compared either with the controls or with PCOS normo-insulinemic women. The authors concluded that IR seems to be more dependent on obesity, while hyperinsulinemia is a primary feature of PCOS [93].

Three important studies in this field have been carried out over the last few years. A study by Amato et al. included 22 women with PCOS and 21 age- and BMI- matched women with prediabetes who were subjected to hyperinsulinemic-euglycemic clamp and OGTT testing. They demonstrated comparable insulin sensitivity between both groups; however, the hyperinsulinism observed in PCOS set both groups apart, and both fasting and glucose-stimulated insulin levels in women with PCOS were strongly influenced by MCRI, which was significantly lower in the PCOS group [31]. Similar relationships between the secretion and degradation of insulin were also demonstrated in a recent study, published in 2020, that included a relatively large group of 190 women with PCOS with normal glucose tolerance. MCRI was impaired in about two-thirds of women with PCOS, and significant relationships were found between MCRI and several clinical, hormonal, and metabolic features of these subjects. In multivariate analysis, the degree of adiposity, estimates of insulin secretion, and serum androgen concentrations were independent predictors of MRCI. Conversely, age, adiposity, MCRI, and insulin sensitivity, but not serum androgens, were independent predictors of insulin secretion [85]. These results indicate that in women with PCOS, excess body fat contributes to hyperinsulinemia through both the increased secretion and reduced clearance of insulin, whereas age and IR modulate insulin secretion, and serum androgens modulate insulin clearance [85]. Contrasting results were presented in a study that included 41 normoglycemic women with PCOS and 68 controls who received a standardized carbohydrate-rich test meal in order to generate a submaximal insulin and glucose stimulation. In PCOS patients, the insulin secretion test showed almost identical baseline and postprandial insulin levels when compared with those of the age- and BMI- matched eumenorrheic controls; however, the baseline and postprandial glucose levels were significantly elevated, as was the C-peptide level. In light of the elevated C-peptide levels, the authors proposed the possibility that insulin levels did not increase more in the test group than it did in the controls group, with increasing glucose levels as indicative of higher insulin clearance in PCOS patients [94]. To the best of our knowledge, this was the first group proposing such a mechanism, and theirs are the only data supporting this hypothesis.

In summary, insulin clearance presents an essential and most often overlooked area of insulin metabolism. Most studies demonstrated its reduced rate in PCOS patients as contributing to hyperinsulinemia, irrespective of the secretion from the beta cells.

## 6. Conclusions

Despite the ongoing progress in our understanding of PCOS as a complex and heterogeneous syndrome and the advances in its management, the question of its primary pathophysiological driver remains unanswered. Although commonly proposed as an initial abnormality, IR is most often looked at in isolation, without the proper investigation of other essential steps in insulin metabolism. In our review, we shed some light on the role of insulin secretion from beta cells and insulin clearance that could offer additional insight into the development of hyperinsulinemia and its relationship to hyperandrogenemia. In general, we can conclude that the available data points out that PCOS patients are more insulin resistant compared to their age- and BMI-matched peers, and IR is also often observed in lean patients. The molecular mechanism behind IR seems to be unique to PCOS, compared to other insulin-resistant states. Beyond the impaired insulin action at the cellular level, there is sufficient evidence for the dysregulation of insulin secretion from beta cells and its reduced hepatic clearance. A detailed analysis of the available literature on insulin metabolism is challenging due to the use of different diagnostic criteria for PCOS, a lack of proper patient phenotyping in study designs, the use of different surrogate estimates of IR, and often, the disregard for the patient’s age and the PCOS duration. The latter, especially, could have a profound effect on understanding insulin metabolism, since different underlying abnormalities could be observed in various stages of the disease process, as seen in T2D.

## Data Availability

Not applicable.
